# The role of the absence of Hyrtl’s anastomosis in monochorionic pregnancy: Friend or foe?: A case report

**DOI:** 10.1097/MD.0000000000033611

**Published:** 2023-05-05

**Authors:** Ziling Liu, Jie Ruan

**Affiliations:** a Department of Obstetrics and Gynecology, Key Laboratory of Birth Defects and Related Diseases in Women and Children of Ministry of Education, West China Second Hospital, Sichuan University, Sichuan, China.

**Keywords:** case report, Hyrtl’s anastomosis, monochorionic diamniotic twin pregnancy, placental territory discordance, selective fetal growth restriction

## Abstract

Hyrtl’s anastomosis, an intra-arterial shunt, plays a protective role when the placental territories supplied by the umbilical arteries are different in size. Its absence is associated with an increased risk of poor outcomes in singleton pregnancy. However, the literature and studies on the effect of absent Hyrtl’s anastomosis in twin placentas are rare.

**Case presentation:**

We present a case of a monochorionic diamniotic twin pregnancy complicated with type I selective fetal growth restriction (SFGR). Despite discordance in placental territory and cord insertion sites, the patient had an overall good pregnancy process, suggesting that the absence of Hyrtl’s anastomosis may have played a benign role.

**Conclusions:**

The absence of Hyrtl’s anastomosis in our case seemed to show a favorable effect, representing a finding of opposite effects in monochorionic placentas compared with singleton placentas.

## 1. Introduction

Hyrtl’s anastomosis, an intra-arterial shunt, is present in approximately 96% of umbilical cords between the umbilical arteries, is usually 1.5 to 2 cm long and positioned within 3 cm of the placental cord insertion.^[1]^ From the limited studies of singleton placentas, the presence of Hyrtl’s anastomosis plays a protective role when the placental territories supplied by the umbilical arteries are different in size.^[2]^ It equalizes pressures between the 2 umbilical arteries before entering the placenta and functions as a safety valve in the event of placental compression or umbilical artery (UA) blockage.^[1]^ In placentas lacking a Hyrtl’s anastomosis, the 2 umbilical arteries supplied a similar area, indicating a relatively high degree of symmetry.^[3]^ However, the literature and studies on Hyrtl’s anastomosis in twin placentas are scarce.

Here, we reported a monochorionic diamniotic twin pregnancy complicated with type I selective fetal growth restriction (SFGR). Despite discordance in placental territory and cord insertion sites, the patient had an overall good pregnancy process, suggesting that the absence of Hyrtl’s anastomosis may have played a benign role.

## 2. Case presentation

**Figure 1.**
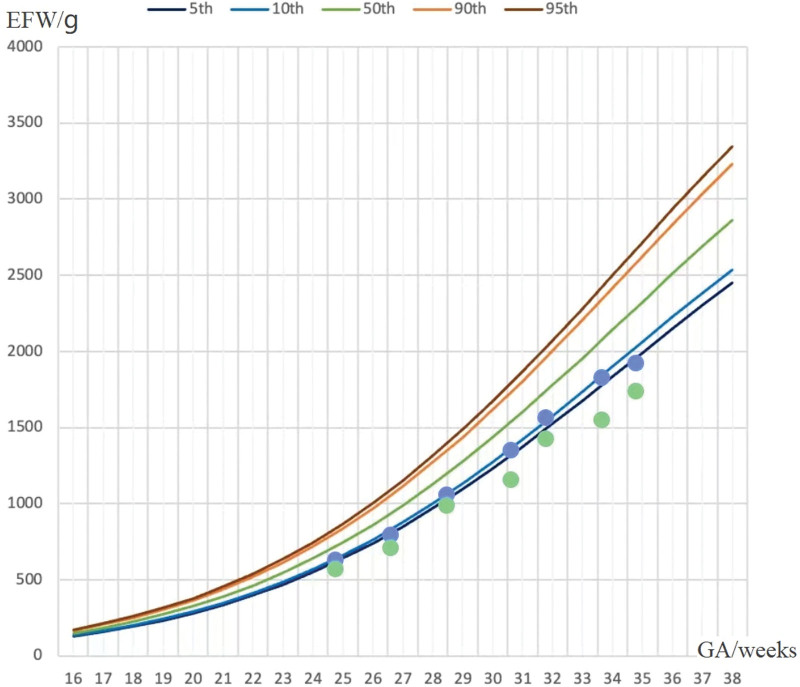
Fetal growth velocity in 2 fetuses (The blue dots represent EFW of the larger fetus at different wk of gestation. The green dots represent EFW of the smaller/CHD fetus at different wk of gestation)* CHD = congenital heart disease, EFW = estimated fetal weight, GA = gestational age.

A 29-year-old woman, gravida 4 para 0, spontaneously conceived. Ultrasound examination at 12 weeks gestation confirmed monochorionic diamniotic twin pregnancy. The patient was referred to our obstetrics unit at 24 + 5 weeks of gestation because of suspected fetal complex congenital heart disease (CHD). Ultrasound in our hospital suggests aortic coarctation associated with ventricular septal defect in one of the twins. At the first visit, the estimated fetal weight of the CHD fetus fell below the 3rd percentile, and discordance between the estimated fetal weight of the 2 fetuses was 9.4%. Therefore, type I SFGR was also diagnosed. Doppler studies of both twins as well as fetal viability were routinely performed at roughly 2-weeks interval. Serial ultrasound monitoring showed normal fetal growth velocity (see in Table 1 and Fig. 1), amniotic volume, UA Doppler, ductus venosus Doppler, and middle cerebral artery Doppler in both fetuses. No significant chromosomal abnormalities in either fetus were detected by chromosome microarray analysis. The patient chose to continue the pregnancy and refused to undergo elective feticide after multidisciplinary consultation with the pediatric cardiovascular physician, neonatologist, and medical geneticist.

**Table 1:** Fetal growth velocity in 2 fetuses.

GA/wk	24^+5^	26^+4^	28^+3^	30^+4^	31^+5^	33^+4^	34^+5^
EFW of the larger fetus/g	646	813	1075	1366	1579	1842	1937
EFW of the smaller fetus/g	585	725	1005	1171	1439	1567	1755
EFW discordance ratio	9.4%	10.8%	6.5%	14.3%	11.8%	14.9%	9.4%

*EFW = estimated fetal weight, GA = gestational age.

The patient was admitted at 30 weeks of gestation for threatened preterm labor and treated with dexamethasone and magnesium sulfate, and discharged at 30 + 4 weeks after inhibition of uterine contractions. At 35 + 3 weeks` gestation, she was readmitted for cervical dilation and underwent an emergency cesarean section. The birth weight of twin female neonates was 2110/2000g (birthweight discordance ratio was 5.2%), and the 1-minute Apgar score was 10. The smaller fetus was immediately transferred to the neonatal intensive care unit and underwent single-stage surgical repair 14 days after birth.

**Figure 2.**
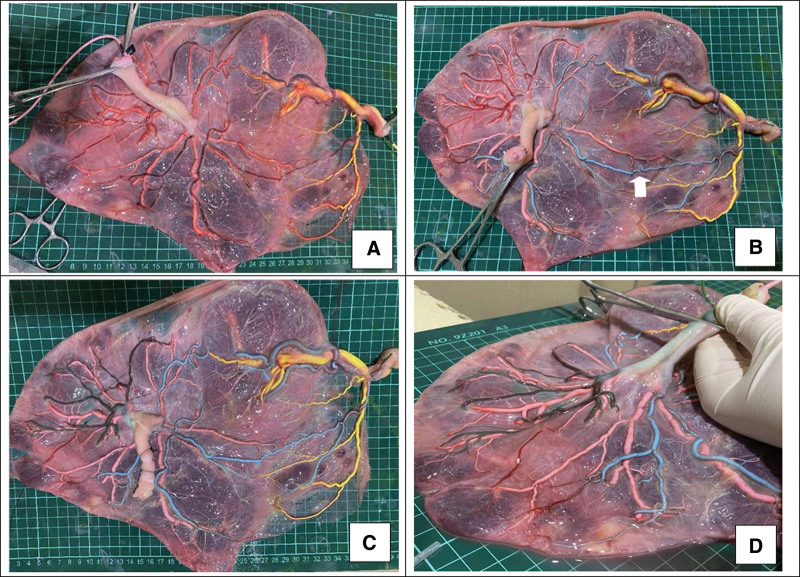
Placental perfusion staining. (A) In the 1st step, the umbilical veins of both fetuses were perfused with yellow dye and pink dye, respectively. (B) In the 2nd step, 1 umbilical artery of the smaller fetus was perfused with blue dye, and blue staining of the branch vessels of the 2 umbilical arteries of the smaller fetus and 1 umbilical artery of the larger fetus through the arterial-arterial anastomosis (white arrow) was observed. (C) In the 3rd step, the other umbilical artery of the larger fetus was perfused with green dye. (D) No color mixing was observed between the 2 umbilical arteries of the larger fetus and their branch vessels).

Placental perfusion staining suggests the placental territory discordance ratio was 64.2% (calculated by Image J software processing) with velamentous cord insertion of the smaller/CHD fetus. The smaller fetus had a placental area of only 13 × 9 cm with an islet-like succenturiate placenta of 3 × 2cm. Rarely, we found a lack of Hyrtl’s anastomosis between 2 umbilical arteries in the larger fetus (see in Fig. 2).

To make comparisons, we also reviewed more than 50 cases of monochorionic placenta stained by perfusion from December 2021 to August 2022 in our hospital and found that a total of 9 cases had placental territory discordance ratio >50% (for more information on the images see Supplementary Files, http://links.lww.com/MD/I853). The remaining 8 patients, except the present case, all presented with type Ⅱ to Ⅲ SFGR. Placental abruption and the smaller fetal demise occurred in 1 case. Considering the poor prognosis of the larger surviving fetus, the patients demand lethal induction of labor. The average gestational age at delivery for the other 7 cases was 31 + 6 weeks, and the average birthweight discordance ratio was 36.2%.

## 3. Discussion and conclusions

In early gestation, multiple anastomoses occur between the umbilical arteries but are subsequently reduced to 1 connection within the first 3 cm of cord proximal to the chorionic plate, that is, Hyrtl’s anastomosis. It was first noted by the descriptive anatomist Joseph Hyrtl, in 1870. The positive effect of this anastomosis in the singleton placentas has been reported in previous researches. The main function is to balance the pressure and flow in the 2 umbilical arteries helping to equalize the area of placental vascular territories. Raio^[2]^ reported a series of antenatal functional evaluations of this anastomosis in 41 women. The difference between the resistance indices of the 2 umbilical arteries was higher after than before the anastomosis. In Byrne’s models,^[4]^ placentas with an anastomosis present showed relatively small discordance between blood pressures feeding each lobe compared with larger differences predicted when an anastomosis was not included. Therefore, Hyrtl’s anastomosis plays a favorable role in equalizing blood pressures between the placental lobes fed by each UA in singleton placentas, especially in the context of compensatory prevention of potential fetal compromise due to placental insufficiency and its absence is associated with an increased risk of poor outcomes.

Studies on Hyrtl’s anastomosis of twin placentas, especially monochorionic placentas, are rare. We finally found only 1 paper on this anastomosis. However, the opposite result compared to the singleton placenta was obtained. Walker^[5]^ reported a case of monochorionic twin pregnancy associated with SFGR and suspected twin-twin transfusion syndrome. Reversed end-diastolic flow, which is an immediate precursor of fetal demise, was noted at multiple sites in the umbilical cord of the smaller/donor twin on the day of hospital admission. Fortunately, the case had a prolonged fetal survival period of 2 weeks before delivery. Pathologic examination of the placenta after delivery disclosed an unbalanced vascular communication between the twins and the absence of Hyrtl’s anastomosis in the smaller/donor twin. The authors believe the absent Hyrtl’s anastomosis can allow adequate fetal oxygenation via the flow through 1 UA despite persistent reversed end-diastolic flow in the other, and allow for the relative independence of interfetal vascular anastomoses to better offset adverse effects of the twin-to-twin transfusion syndrome.

The absence of Hyrtl’s anastomosis in our case also seemed to show a favorable effect similar to this literature above. However, the difference is that the absent Hyrtl’s anastomosis appears in the larger twin, not the smaller twin. In comparison to the other monochorionic placentas with large placental territory discordance, the growth difference between the twins in this case gradually decreased with increasing gestational weeks and consistently maintained good umbilical blood perfusion. The possible reason is that the 2 umbilical arteries of the larger fetus do not interfere with each other. Branches of 1 UA coincide with the smaller fetus to compensate for the relatively small placental volume, and the other UA is independent to ensure adequate cotyledonary perfusion and adequate fetal oxygenation of the larger fetus. The absence of an equalizing anastomosis allows 2 relatively separate and independent placental circulations. Thus, it is like transforming the placenta into an intermediate state between dichorionic and monochorionic.

In conclusion, this case represents a finding of opposite effects of Hyrtl’s anastomosis in monochorionic placentas compared with singleton placentas. More cases of placental perfusion staining are needed to support our view.

## Author contributions

**Formal analysis:** Jie Ruan.

**Investigation:** Jie Ruan.

**Methodology:** Jie Ruan.

**Writing – original draft:** Liu Ziling.

**Writing – review & editing:** Liu Ziling.

## Supplementary Material


