# Supplementary material for: The role of the absence of Hyrtl’s anastomosis in monochorionic pregnancy: Friend or foe?: A case report
Source: Medicine (Baltimore). 2023 May 5;102(18):e33611. doi: 10.1097/MD.0000000000033611 (PMC10158883; doi:10.1097/MD.0000000000033611)
Supplement: Supplementary file 1 [file medi-102-e33611-s001.pdf]

Supplementary Files: Stained monochorionic diamniotic placentas with large territory discordance in our hospital from Dec.2021 to Aug. 2022.

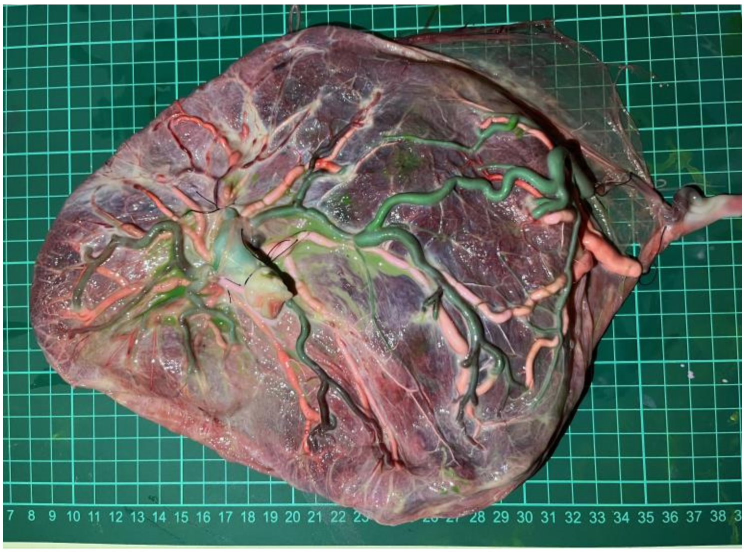	Case 1#<br>Cord insertion: normal/velamentous<br>Placental territory discordance ratio:76.5%<br>Complication: SFGR (type □)<br>GA at delivery: 33+1GW<br>Twin birth weight: 2060/1390g<br>Birth weight discordance ratio: 32.5%
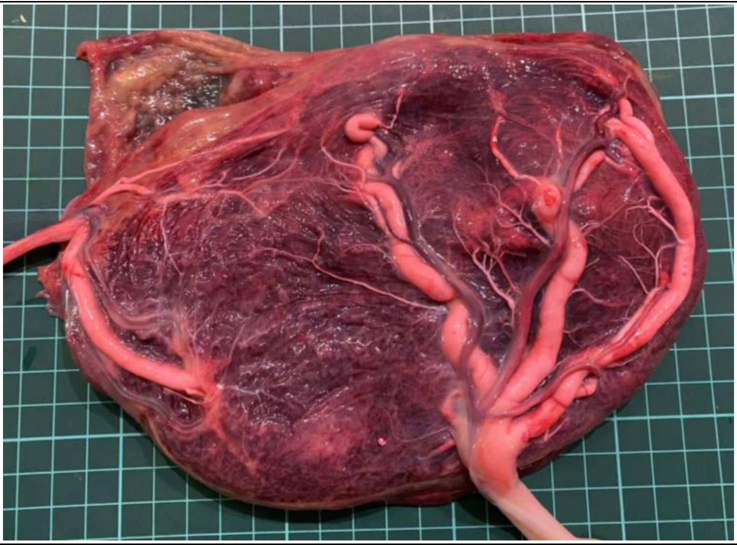	Case 2#<br>Cord insertion: marginal/marginal<br>Placental territory discordance ratio:52.5%<br>Complication: SFGR (type □)<br>GA at delivery: 30+6GW<br>Twin birth weight: 1330/670g<br>Birth weight discordance ratio: 49.6%
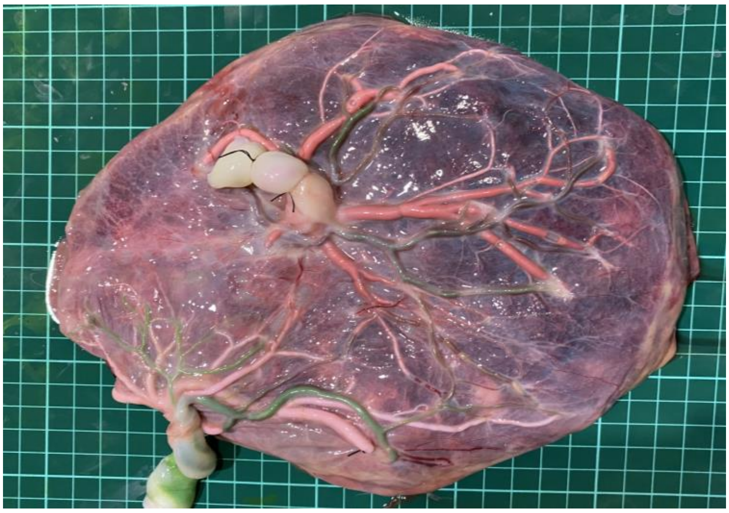	Case 3#<br>Cord insertion: normal/marginal<br>Placental territory discordance ratio:60.7%<br>Complication: SFGR (type □)<br>GA at delivery: 32+6GW<br>Twin birth weight: 1660/1280g<br>Birth weight discordance ratio: 22.9%

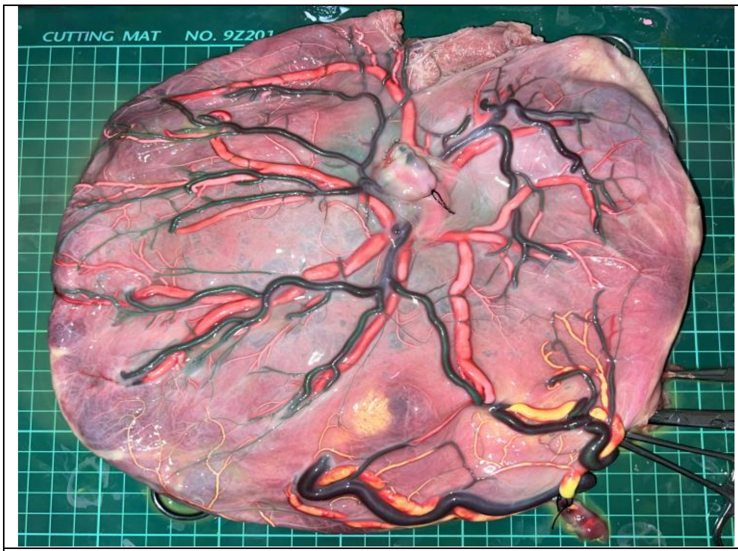	Case 4#<br>Cord insertion: normal/marginal<br>Placental territory discordance ratio: 65.9%<br>Complication: SFGR(type□), TTTS(stage□)<br>GA at delivery: 28+1GW<br>Twin birth weight: 1380/790g<br>Birth weight discordance ratio: 42.8%
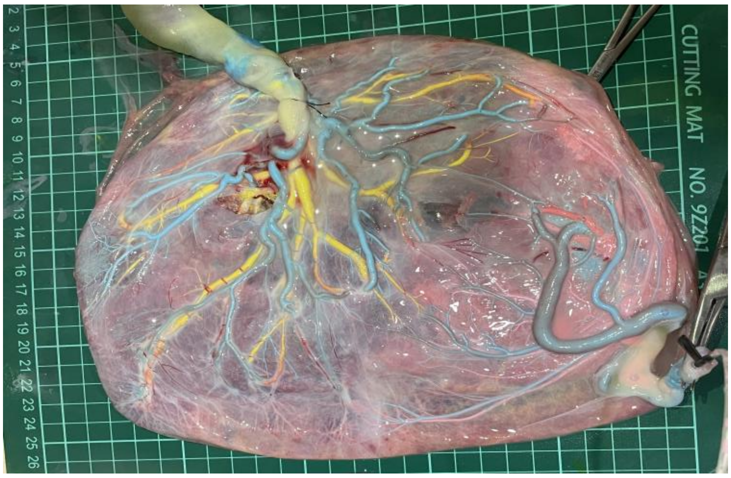	Case 5#<br>Cord insertion: normal/marginal<br>Placental territory discordance ratio: 50.5%<br>Complication: SFGR (type □)<br>GA at delivery: 32+2GW<br>Twin birth weight: 1710/970g<br>Birth weight discordance ratio: 43.3%
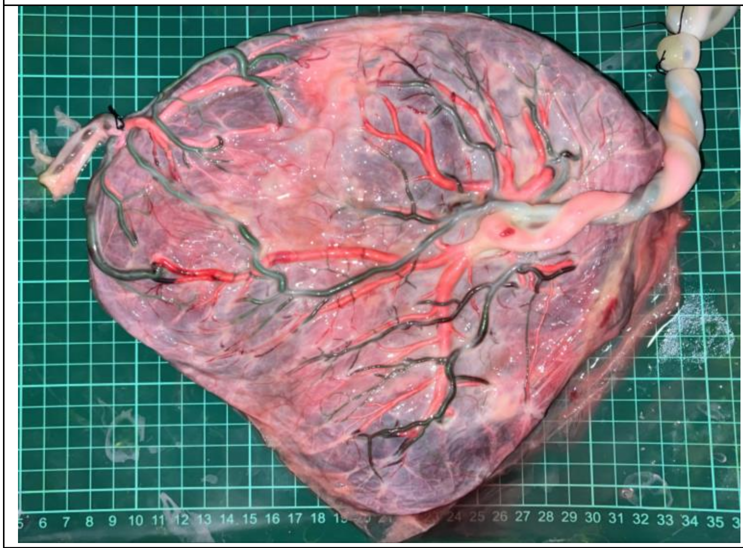	Case 6#<br>Cord insertion: normal/marginal<br>Placental territory discordance ratio: 69.8%<br>Complication: SFGR (type □)<br>GA at delivery: 33+1GW<br>Twin birth weight: 2060/1300g<br>Birth weight discordance ratio: 36.9%

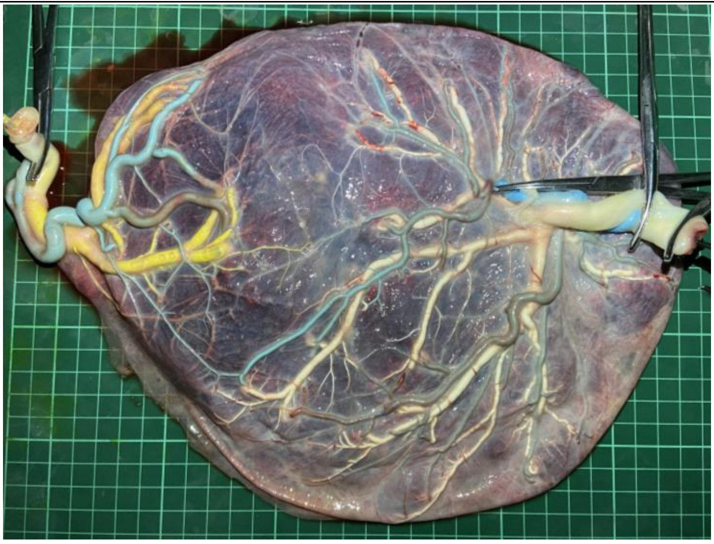	Case 7#<br>Cord insertion: normal/marginal<br>Placental territory discordance ratio: 66.9%<br>Complication: SFGR (type □)<br>GA at delivery: 33+6GW<br>Twin birth weight: 2100/1650g<br>Birth weight discordance ratio: 21.4%
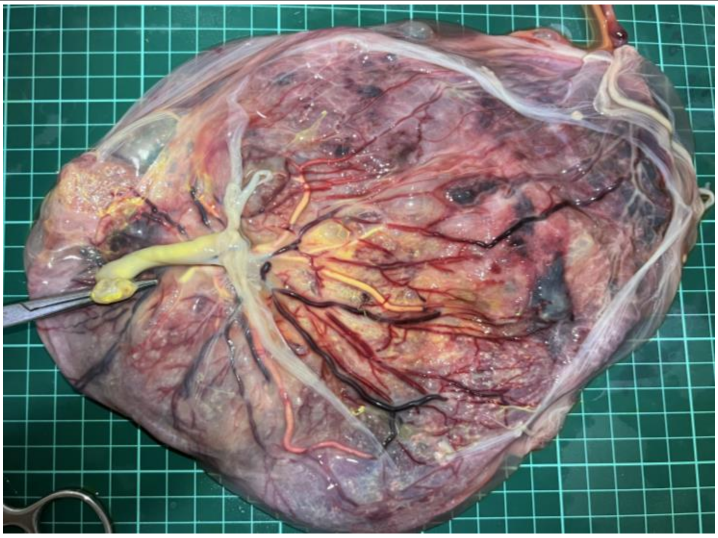	Case 8#<br>Cord insertion: normal/marginal<br>Placental territory discordance ratio: 81.3%<br>Complication: SFGR(type□), TTTS(stage□)<br>GA at delivery: 23+1GW<br>Twin birth weight: 571/349g<br>Birth weight discordance ratio: 38.9%

* GA: gestational age; SFGR: selective fetal growth restriction; TTTS: twin-twin transfusion syndrome.
